# Distinct immunological activation profiles of dSLIM® and ProMune® depend on their different structural context

**DOI:** 10.1002/iid3.126

**Published:** 2016-10-18

**Authors:** Kerstin Kapp, Jacqueline Schneider, Lisa Schneider, Nadine Gollinge, Stefanie Jänsch, Matthias Schroff, Burghardt Wittig, Christiane Kleuss

**Affiliations:** ^1^ Mologen AGBerlinGermany; ^2^ Foundation Institute Molecular Biology and BioinformaticsFreie Universitaet BerlinBerlinGermany

**Keywords:** Cancer immunotherapy, plasmacytoid dendritic cells, structure‐function relationship

## Abstract

**Introduction:**

DNA‐based TLR9 agonists are potent activators of the immune system. ProMune® and dSLIM® belong to different families of TLR9 agonists and both have been established as cancer immunotherapeutics in clinical proof‐of‐concept studies. Unfortunately, ProMune® failed in pivotal oncological trials. dSLIM®, the active ingredient of Lefitolimod (MGN1703), successfully finished a double‐blinded, placebo‐controlled phase II study in patients with advanced colorectal cancer, exhibiting improved progression‐free survival and durable disease control.

**Methods:**

To explain the different systemic efficacies of dSLIM® and ProMune®, both TLR9 agonists and chimeric molecules thereof are analyzed side‐by‐side in a panel of in vitro assays for immune activation.

**Results and conclusions:**

Indeed, dSLIM® exposure results in an IFN‐α dependent broad activation of immune cells whereas ProMune® strongly stimulates B cells. Moreover, all functional effects of dSLIM® strictly depend on the presence of CG‐motifs within its dumbbell‐shaped, covalently closed structural context. Conversely, several immunological effects of ProMune® like IL‐8 secretion are independent of CG‐motifs and could be ascribed to the phosphorothioate‐modifications of its DNA backbone, which may have caused the side effects of ProMune® in clinical trials. Finally, we showed that the implementation of ProMune® (ODN2006) base sequence into the characteristic dSLIM® dumbbell form resulted in dSLIM2006 with all beneficial effects for immunostimulation combined from both TLR9 classes without any CG‐independent effects.

## Introduction

Recently, cancer fighting strategies utilizing the human immune system came into focus. Here, the body's intrinsic ability to discriminate cancer cells from regular body cells and to destroy them is employed as a therapeutic principle. In general, it is assumed that initially elicited innate and adaptive immune responses against malignant cells were then blunted either by auto‐regulatory mechanisms of the immune system, or through evolved immune escape strategies of the cancer cells themselves or their hosting cellular niches 1.

The 10 members of human Toll‐like receptors (TLR) recognize different groups of pathogen‐ or damage‐associated molecular pattern (PAMP, DAMP) and initiate a strong activation cascade of the innate and adaptive immune systems 2, 3, 4. Bacterial and viral DNA, but also mitochondrial DNA as well as synthetic oligodeoxynucleotides (ODN), all containing non‐methylated CG‐motifs, were identified as PAMP and DAMP molecules 4 with a wide range of powerful immunological activities 5, 6, 7, 8, 9. The cellular responses to DNA containing non‐methylated CG‐motifs are mediated by TLR9 10. Although in primates strong stimulation of TLR9 on B lymphocytes (B cells) and plasmacytoid dendritic cells (pDC) can be achieved by using certain ODN in the absence of CG‐motifs 11, all TLR9 agonists hitherto accepted for clinical development contain several CG‐motifs. TLR9 is localized to intracellular compartments and detects DNA in the endolysosomal compartment 12. The host's DNA is regularly not accessible in these compartments, and therefore does not activate TLR9. Exceptions are cellular injuries, leading to the release of nuclear and mitochondrial content to the cytoplasmic compartment or extracellular space, making them accessible for autophagy or cellular re‐uptake and subsequent TLR9 binding. Especially, mitochondrial DNA represents an endogenous damage associated molecular pattern molecule 9, 13 as mitochondria evolved from bacteria over endosymbionts to organelles with their DNA still showing certain features of bacterial DNA, like non‐methylated CG‐motifs.

TLR9 has a rather limited expression profile in humans compared to other TLR, and is exclusively expressed in resting human immune cells by pDC and B cells 2, 14. Intracellular signaling triggered by TLR9 activation results in up‐regulation of two pathways: (1) the activity of the nuclear factor kappa‐light‐chain‐enhancer of activated B cells (NFκB) is triggered for the production of pro‐inflammatory cytokines and acquisition of antigen‐presenting functions; and (2) activation of interferon (IFN) regulatory factor 7 (IRF7) leads to type I IFN production 15, 16. IFN‐α is central to link the stimulated innate response to the adaptive arm of the immune system 17 conferring also tumor antigen specific cellular and humoral immune responses.

Due to the unique TLR9 detection profile and the triggered immune responses, TLR9 agonists are currently being tested as adjuvants of antimicrobial, anti‐allergic, and especially anticancer immunotherapy. Sequences, early identified by the pioneers in pre‐clinical and clinical research on TLR9 agonists Yamamoto, Klinman, Krieg, or Raz 5, 6, 7, 8, were further developed, and phosphorothioate (PTO) modifications were introduced into molecules. Nucleic acid modification by PTO was supposed to prevent degradation by exonucleases and along other strategies was already in use for antisense therapeutics 18. The resulting TLR9 agonists were assigned to classes with distinct structural and biological characteristics: A‐class CpG‐ODN (also known as D‐type) 19, 20, B‐class CpG‐ODN (also known as K‐type) 20, 21, C‐class CpG‐ODN 22, 23, P‐class CpG‐ODN 24. A new family of TLR9 agonists is represented by dSLIM® 25, which is a dumbbell‐shaped, covalently closed molecule without any PTO modification.

For detailed comparison regarding their immunomodulatory properties we have picked the structurally and chemically different TLR9 agonists dSLIM® and ProMune®. dSLIM^®^ is the only TLR9 agonist in clinical development for monotherapy that consists exclusively of natural nucleotides. It has recently successfully finished a double‐blinded phase II study in patients with advanced colorectal cancer, exhibiting improved progression‐free survival, and durable disease control, compared to placebo 26. ProMune® (B‐class) is prominent for being the first TLR9 agonist undergoing human studies and exhibiting exceptional immune activation both in vitro and in vivo 27. Unfortunately, the in vivo efficacy of ProMune® was compromised by a narrow clinical therapeutic window, which finally led to premature closure of advanced oncological trials 28. The linearly configured ProMune® uses PTO‐modification in its nucleotides to stabilize the DNA against exonuclease degradation. In mice, these artificial nucleotides and the resulting modified DNA backbone at high concentrations dramatically altered morphology and functionality of lymphoid organs, induced hemophagocytic lymphohistiocytosis, and macrophage activation syndrome 29, 30. In monkeys, repeated and long‐timed application of PTO‐ODN resulted in improper complement activation 31 and inhibition of blood coagulation 32, 33.

Here, we dissect the different activation profiles of dSLIM® and ProMune®, and trace back the adverse effects of ProMune® to the mere presence of PTO‐modifications in the agonist structure. Furthermore, PTO‐modification of highly potent TLR9 agonists may even blunt their activation efficacy on immune cells. Otherwise, if the sequence of bases of ProMune® was introduced as natural nucleotides into the structurally stabilized environment of dSLIM®, the resulting dSLIM® molecule dSLIM2006‐PD (phosphorodiester, Fig. S5) strongly activated the relevant immune cells in a CG‐motif‐dependent manner without any toxic effects detectable at the cellular level.

## Materials and Methods

### Sequences of TLR9 agonists

**Figure 1:**
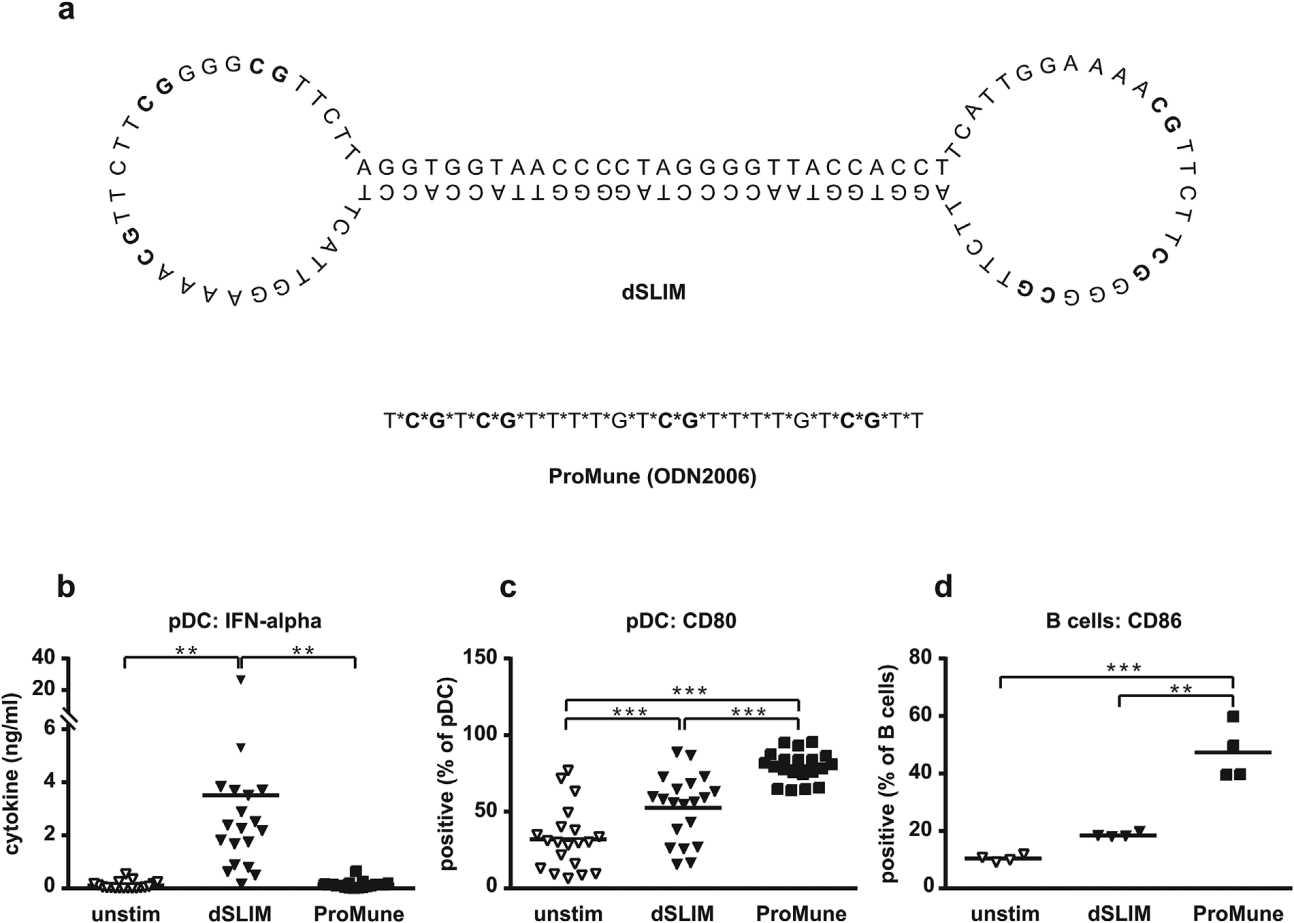
Structure and functions of dSLIM® and ProMune® on TLR9 positive cells. (a) Sequences and proposed structures of dSLIM® and ProMune®. CG‐motifs are depicted in bold. *Indicate a PTO bond instead of genuine phosphodiester bond between deoxynucleotides. (b–d) Isolated TLR9‐positive cells, pDC, and B cells, were treated with dSLIM® or ProMune® at final concentrations of 3 μM or medium alone for 48 h. A final concentration of 10 ng/mL IL‐3 was added to the pDC cultures. IFN‐α levels in the supernatants of pDC (b) were determined by a bead‐based multiplex immunoassay (*n* = 19, means are shown, **P < 0.01, repeated measures ANOVA, Bonferroni's multiple comparison test). pDC were stained with αCD80 antibody and analyzed by flow cytometry (c). Frequency of CD80‐expressing cells within the pDC population are shown (*n* = 20, means are shown, ***P < 0.001, repeated measures ANOVA, Bonferroni's multiple comparison test). B cells (d) were stained with αCD86 antibody and analyzed by flow cytometry. Frequency of CD86‐expressing cells within the B cell population are shown (*n* = 4, means are shown, **P < 0.01, ***P < 0.001, repeated measures ANOVA, Bonferroni's multiple comparison test).

dSLIM® and ProMune® as depicted in Figure 1. dSLIM®: educt ODN for dSLIM®, CTAGGGGTTACCACCTTCATTGGAAAACGTTCTTCGGGGCGTTCTTAGGTGGTAACCC; educt for dSLIM2006‐PD, CTAGGGGTTACCACCTTCATCGTCGTTTTGTCGTTTTGTCGTTCTTAGGTGGTAACCC; educt for dSLIM2006‐PTO, CCTAGGGGTTACCACCTTCAT*C*G*T*C*G*T*T*T*T*G*T*C*G*T*T*T*T*G*T*C*G*T*TCTTAGGTGGTAACC; educt for dSLIM2006‐PD(‐CG), CTAGGGGTTACCACCTTCATGCTGCTTTTGTGCTTTTGTGCTTCTTAGGTGGTAACCC; educt for dSLIM2006‐PTO(‐CG), CCTAGGGGTTACCACCTTCAT*G*C*T*G*C*T*T*T*T*G*T*G*C*T*T*T*T*G*T*G*C*T*TCTTAGGTGGTAACC; LMLS variants: LMLS, TCATTGGAAAACGTTCTTCGGGGCGTTCTT; LMLS‐1tPTO, T*CATTGGAAAACGTTCTTCGGGGCGTTCT*T; LMLS‐2tPTO T*C*ATTGGAAAACGTTCTTCGGGGCGTTC*T*TT; LMLS‐PTO, T*C*A*T*T*G*G*A*A*A*A*C*G*T*T*C*T*T*C*G*G*G*G*C*G*T*T*C*T*T; LMLS‐PTO(‐CG), T*C*A*T*T*G*G*A*A*A*A*G*C*T*T*C*T*T*T*G*G*G*G*G*C*T*T*C*T*T; ProMune‐CG, T*G*C*T*G*C*T*T*T*T*G*T*G*C*T*T*T*T*G*T*G*C*T*T; ODN2216, G*G*GGGACGATCGTCG*G*G*G*G*G denotes PTO bond between deoxynucleotides. ODN were custom synthesized by Micro­synth (Balgach, Switzerland) or TIB Molbiol (Berlin, Germany). Construction of dSLIM® is described elsewhere 25, dSLIM® variants are constructed accordingly.

### Cell isolation and activation

Buffy coats from anonymized healthy donors were obtained from the “DRK‐Blut­spende­dienst—Ost” (Berlin, Germany). PBMC were isolated by density gradient centrifugation using Ficoll (Biochrom, Berlin, Germany; now Merck Millipore). pDC were prepared using the Human Diamond pDC Isolation Kit (Miltenyi Biotec, Bergisch Gladbach, Germany) according to the manufacturer's instructions. B cells were isolated from PBMC using the CD19 MicroBead Kit (Miltenyi Biotec).

Cells were cultured in complete medium (RPMI1640 [Lonza, Basel, Switzerland] with 2 mM UltraGlutamine [Lonza] supplemented with 10% (v/v) fetal calf serum [Linaris, Dossenheim, Germany], 100 U/mL Penicillin and 100 μg/mL Streptomycin) in flat‐bottom plates (six million PBMC/mL, two million B cells/mL) or 96‐well round bottom plates (0.25 million pDC/mL, in the presence of 10 ng/mL recombinant IL‐3 [PeproTech, Hamburg, Germany]). B18R (eBioscience, Frankfurt/M, Germany), a vaccinia virus‐encoded receptor with specificity to type I interferons, was used at a final concentration of 0.5 μg/mL.

### Cell staining and flow cytometry

Cells were surface‐stained with monoclonal antibodies in PBS containing 10% (v/v) human serum, 2.5% (v/v) FCS, and 0.1% (w/v) azide on ice. The following antibodies were used: anti‐lineage cocktail 1, αCD123 (7G3), αHLA‐DR (L243), αCD40 (5C3), αCD11c (B‐ly6), αCD86 (2331 FUN‐1), αCD19 (4G7), αCD69 (FN50), all from BD Biosciences (Heidelberg, Germany) and αCD14 (61D3), αCD169 (7–239), αCD3 (OKT‐3), αCD56 (MEM188), αCD80 (2D10), all from eBioscience.

All flow cytometric parameters of cells were acquired on a FACSCalibur (BD Biosciences). Frequencies were related to the indicated parent populations; geometric means of fluorescent cells were indicated as mean fluorescent intensity (MFI). Data were analyzed with the FlowJo software (Tree Star, Ashland, OR).

### Determination of cytokine concentrations

Secreted cytokines were accumulated in cell growth medium for 2d if not indicated otherwise. ELISA for IFN‐α (eBioscience), IFN‐γ (OptEIA Human IFN‐γ ELISA Set, BD Biosciences), IP‐10, IL‐8, and MCP‐1 (all from R&D Systems, R&D Systems, Wiesbaden‐Nordenstadt, Germany) were performed in duplicates according to the manufacturer's instructions. Optical density was measured at 450 nm; the data were analyzed with the MicroWin software (Berthold Technologies, Bad Wilbad, Germany).

Alternatively, cytokine levels in the cell growth medium were determined in duplicates by a bead‐based multiplex immunoassay (FlowCytomix from eBioscience) according to the manu­fac­turer's instructions. Data were acquired on a FACSCalibur and evaluated with the FlowCytomixPro software (eBioscience). The ELISA as well as the multiplex assay for IFN‐α were specified to detect IFN‐α2a, IFN‐α2b, IFN‐α2c, but not IFN‐α1.

### Cytotoxicity assay

Jurkat cells (DSMZ, Braunschweig, Germany) were used as target cells and labeled for 5 min at 37°C in RPMI1640 (Lonza) with the lipophilic dye Dil (Invitrogen/Life Technologies, Darmstadt, Germany) at a final concentration of 1 μM. After 18 h, 50,000 target cells were co‐cultured with effector cells (treated PBMC) in three different ratios (effector:target 20:1, 10:1, and 5:1). For positive control, PBMC were cultured in 2000 U/mL IL‐2 (PeproTech). Cultures were performed in complete medium and were run for 4 h at 37°C in 96‐well round bottom plates in duplicates. Subsequently, cells were stained with 7‐AAD (BD Biosciences). On a FACSCalibur (BD Biosciences) 5000 target cells were acquired. The percentage of dead target cells was determined from 7‐AAD‐positive cells and specific cytotoxicity calculated:

$$Specific\;cytotoxicity\;(\%)=((\%7-{AAD}^{+}\;of\;\;{Dil}_{induced}^{+})-\left(\%7-{AAD}^{+}\;of\;{Dil}_{spon\,\tan eous}^{+}\right))/(100\%-\%7-{AAD}^{+}\;of\;{Dil}_{spon\,\tan eous}^{+})\times 100$$

### Statistical analysis

Data were analyzed with GraphPad Prism 6 (GraphPad Software Inc., La Jolla, CA, USA). The paired data were analyzed with the repeated measures ANOVA test. Depending on the complexity and hypothesis for the individual experiment, the following post tests were used: Bonferroni's and Dunnetts multiple comparison tests as well as the Fisher's LSD test. The utilized post tests are indicated in the figure legends. *P* values <0.05 were considered significant.

## Results

### Comparison of dSLIM® and ProMune®

dSLIM® and ProMune® are DNA molecules, representing different families of TLR9 agonists. Although both molecules display CG‐motifs for binding and activating TLR9, their chemical natures as well as structural and conformational appearances are different (Fig. 1a). dSLIM® is a covalently closed, dumbbell‐shaped DNA‐molecule devoid of any non‐natural modification. Stability against degradation by extra‐ and intracellular exonucleases is conveyed by its circular conformation. In each dumbbell loop, dSLIM® comprises three CG‐motifs separated by five and three nucleotides 25. In contrast, ProMune® is a linear, single‐stranded molecule with the 24 nucleotides linked exclusively by PTO bonds conferring exonuclease‐resistance to the molecule. ProMune® presents four CG‐motifs spaced by one, six, and six nucleotides.

Both molecules change the activation status of TLR9‐positive pDC and B cells, but with a different outcome. dSLIM® is a strong stimulator of IFN‐α release from isolated pDC while ProMune® only marginally activate this intracellular pathway (Fig. 1b). Both molecules up‐regulate the activation marker and co‐stimulatory molecule CD80 on pDC, with ProMune® doing this significantly better than dSLIM® (Fig. 1c). Isolated B cells are strongly activated upon exposure to ProMune® as indicated by the expression of the co‐stimulator CD86, while dSLIM® up‐regulates CD86 on B cells to a lesser extent (Fig. 1d).

**Figure 2:**
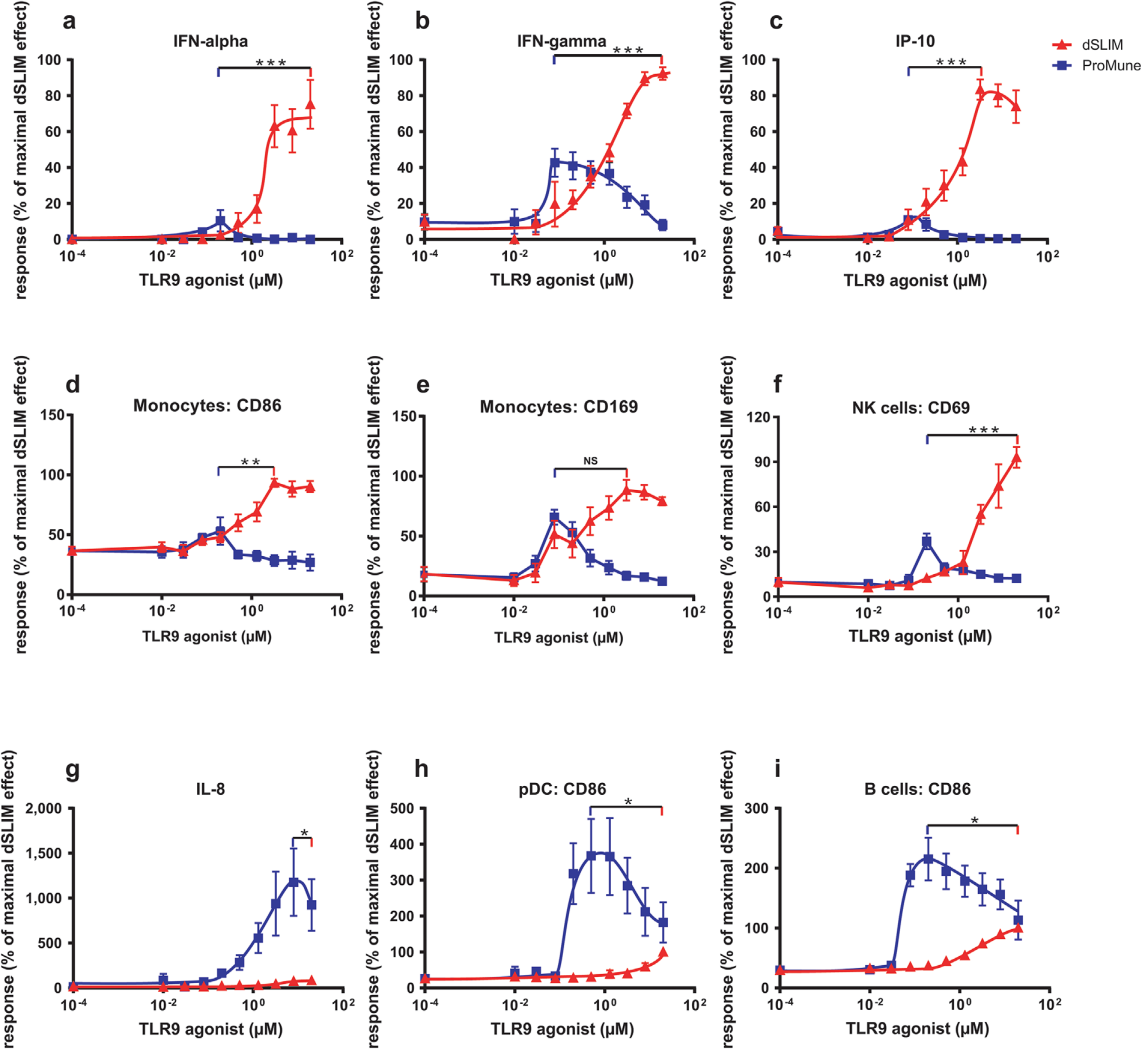
Concentration dependent activation of PBMC. PBMC were treated with dSLIM® or ProMune® at the indicated final concentrations for 48 h. (a–c and g) Cytokine secretion. Cytokine levels in the supernatants were determined by a bead‐based multiplex immunoassay or ELISA. For each cytokine concentration, the analyzed effect was normalized to the effect observed at the maximal effective dSLIM® concentration within the test series (IFN‐α 37–1780 pg/mL; IFN‐γ 74–1100 pg/mL; IP‐10 19,275–290,300 pg/mL; IL‐8 560–180,000 pg/mL). Means and SEM resulting from the stated numbers of individual experiments (a: IFN‐α *n* ≥ 5, b: IFN‐γ *n* ≥ 5, c: IP‐10 *n* ≥ 5, g: IL‐8 *n* ≥ 5) are shown. (d–f and h, i) Expression of activation markers within selected cell populations. PBMC were stained with antibodies against lineage‐ and activations markers and analyzed by flow cytometry as described in Materials and Methods section. For each analyte the analyzed MFI or the frequency of activation markers within the gated cell popuation was normalized to the expression level obtained with the most effective dSLIM® concentration (monocytes CD86 MFI: 89.5–165; monocytes CD169 MFI: 491–1371; frequency of CD69‐expressing cells in the NK cell population: 1.51–21.8%; pDC CD86 MFI: 46–570; B cells CD86 MFI: 15.5–26.5). Means and SEM resulting from the stated numbers of individual experiments (CD86 of monocytes, CD169 of monocytes, CD86 of B cells *n* ≥ 3; CD86 of pDC *n* ≥ 2; CD69 of NK cells *n* ≥ 4) are shown. The unpaired *t*‐test was used to analyze the differences between the maximal effects for dSLIM® and ProMune® (*P < 0.05, **P < 0.01, ***P < 0.001, NS, not significant).

Also in the more physiological context of PBMC (Fig. 2) the activation patterns of dSLIM® and ProMune® are distinct: regarding the anti‐tumor activities investigated at the molecular level over the analyzed concentration range from 0.01 to 20 μM dSLIM® predominates with stronger responses than ProMune® (Fig. 2a–f) over a time range of 2 days (Fig. S6). The activation pattern of ProMune® prevails only in distinct immunological features (Figs. 2g–i and S6). Figure 2a shows for dSLIM® an efficient induction of IFN‐α secretion in human PBMC. This is most likely due to direct activation of pDC as already shown on isolated pDC in Figure 1b. IFN‐α activates monocytes 34, 35—indicated by up‐regulation of CD169 and CD86 (see Fig. 2d and e), and IP‐10 release (see Fig. 2c)—as well as natural killer cells 17—indicated by their up‐regulation of CD69 (see Fig. 2f), and IFN‐γ release (see Fig. 2b). ProMune® does only marginally induce IFN‐α secretion from pDC and consequently hardly activates natural killer cells or monocytes (see Figs. 2d–f and S6). ProMune® induces IFN‐γ release also less efficacious than dSLIM®. However, ProMune® heavily stimulates secretion of IL‐8 while a dSLIM® effect on this angiogenesis‐promoting cytokine is barely detectable (Fig. 2g). Although ProMune® hardly activates pDC to release IFN‐α, it activates pDCs to upregulate surface proteins CD80 (see Fig. 1b) and CD86 (see Fig. 2h) more pronounced than dSLIM®. B cells are also better activated upon ProMune® exposure compared to dSLIM® incubation as indicated by CD86 up‐regulation on isolated B cells (Fig. 1d) as well as B cells in the context of PBMC (Figs. 2i and S6).

All these effects of dSLIM® on immune cells, specified either by surface marker expression or cytokine release, are continuously rising with the dSLIM® concentration applied and plateau for most responses at about 10 μM dSLIM® (see Fig. 2a–f). In contrast, biphasic responses are detected for ProMune®: cytokine secretions (IFN‐α, IFN‐γ, IP‐10) are already induced at low levels of 100 nM ProMune®, but decline at higher concentrations. Also cellular activation markers CD86 on pDC and B cells are precipitously up‐regulated from 200 nM up to 1 μM ProMune®; but higher concentrations elicit less CD86 expression. One exception is the stimulation of IL‐8 secretion by ProMune® that continuously rises with increasing ProMune® concentrations up to about 10 μM. In contrast, equimolar concentrations of dSLIM® barely release angiogenic and inflammatory IL‐8.

Activation of NK cells by dSLIM® (see Fig. 2f) profoundly raises their cytotoxic activity against target cells. Table 1 shows that in PBMC dSLIM® activates effector cells to lyse Jurkat cells at different effector‐to‐target ratios. The dSLIM®‐evoked cytotoxicity is significant at relevant concentrations agonist applied, and ranges between 20% and 40% of the maximal cytotoxicity achieved in this experimental setup by treatment with IL‐2. In contrast, PBMC pre‐incubated with 3 μM ProMune® do not exert significant NK cell‐mediated cytotoxic effects; only at concentrations as low as 0.2 μM ProMune®, that we showed in Figure 2f to best activate NK cells (see), significant cytotoxicity ranging between 30% and 40% of the maximum IL‐2 induced cytotoxicity could be detected.

**Table 1:** Induction of NK‐cell‐mediated cytotoxicity in PBMC treated with dSLIM® or ProMune®

Specific cytotoxicity^a^	Activator	μM^b^	Mean^c^	SEM	*n*
Effector:target: 10:1	ProMune®	0.2	34.7	3.4	4
	ProMune®	3	5.8	3.6	8
	dSLIM®	3	26.6	3.8	8
	dSLIM®	20	21.2	11	4
Effector:target: 5:1	ProMune®	0.2	39.7	1.7	4
	ProMune®	3	2.1	2.1.	6
	dSLIM®	3	28.3	8.8	6
	dSLIM®	20	36.5	6.9	4

^a^ After 48 h stimulation with the indicated TLR9 agonist, PBMC were co‐cultured with the indicated proportions of Dil‐labeled Jurkat cells as targets for 4 h. After staining with 7‐AAD, specific cytotoxicity was estimated based on the frequencies of 7‐AAD positive target cells as described in Materials and Methods section.

^b^ PBMC were treated with dSLIM® or ProMune® at the indicated concentrations; 0.2 μM ProMune® and 20 μM dSLIM® were chosen according to the maximal activation of NK cells detected in PBMC under these conditions as shown in Figure 2.

^c^ Means were derived from the calculated specific cytotoxicities after correction for the spontaneous cytotoxicity determined in the negative controls (PBMCs incubated in complete medium) and normalization to the positive controls (PBMC treated with 2000 U/mL recombinant IL‐2).

**Figure 3:**
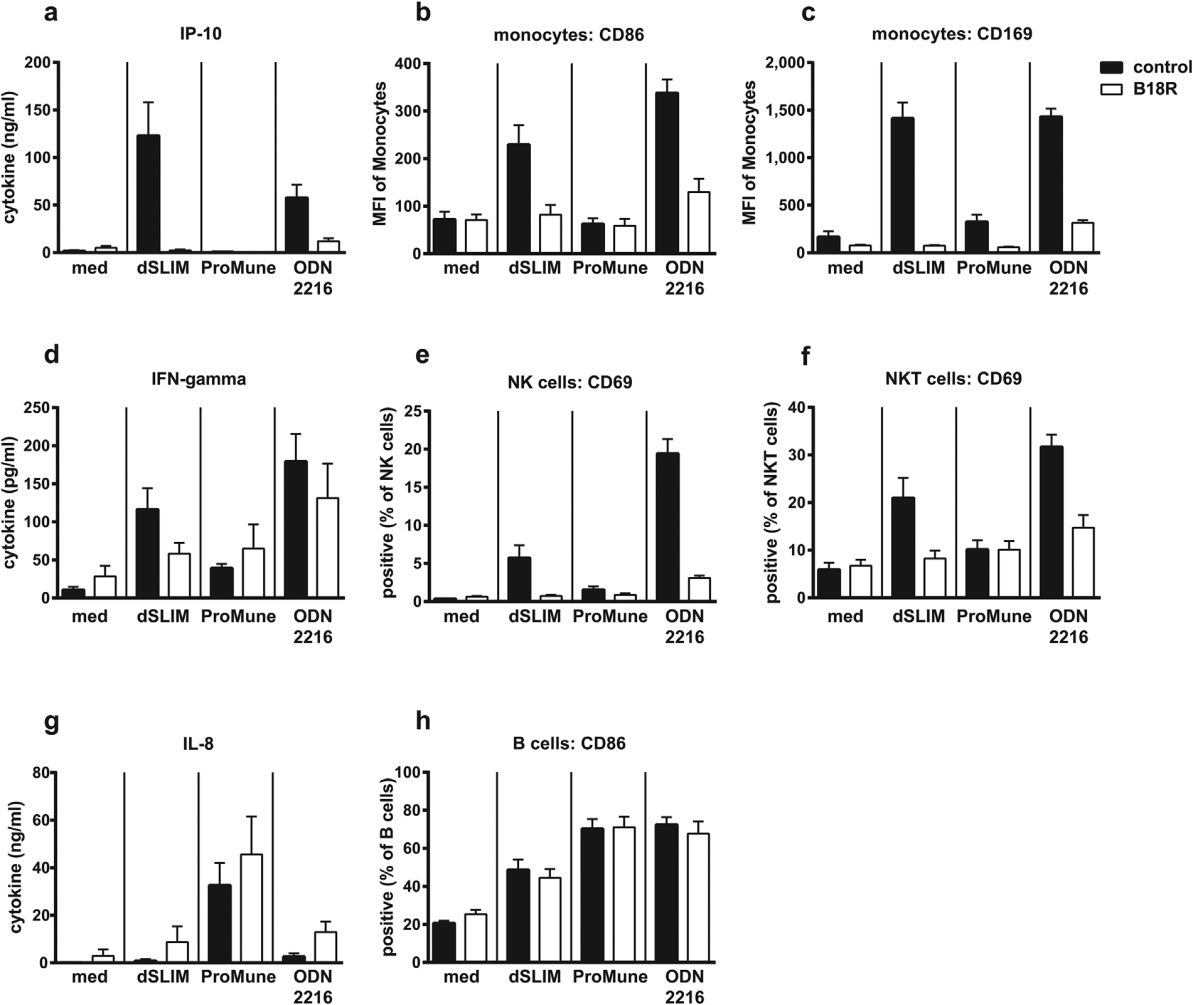
Dependency of dSLIM® and CpG‐ODN effects on type I interferon. PBMC were treated with the TLR9 agonists dSLIM®, ProMune®, or class A CpG‐ODN (ODN2216) at final concentrations of 3 μM or medium alone for 48 h. To block type I interferons, cell cultures were co‐incubated with B18R, a vaccinia virus‐encoded receptor with specificity to type I interferons, at 0.5 μg/mL final concentration (open bars). Cytokine levels in the supernatants were determined by a bead‐based multiplex immunoassay or ELISA. Cells were stained with antibodies against lineage‐ and activation markers and analyzed by flow cytometry as described in Materials and Methods section. Frequencies or MFI of activation markers within the cell population are shown. Means and SEM resulting from four individual experiments are displayed.

While dSLIM® activation of human immune cells depends on TLR9‐activating CG‐motifs 25, ProMune® effects on immune cells seem to be rather unspecific as IL‐8 secretion is completely CG‐independent over a dose range from 0.1 to 8 μM (Fig. S1b). A partial CG‐dependency is observed on B cells upon stimulation with more than 1 μM ProMune®, while in monocytes a weak stimulation is only detectable and partially CG‐dependent at submicromolar concentrations of ProMune® (Fig. S1). Interestingly, at higher concentrations of ProMune® the TLR9‐negative monocytes are directly and CG‐independently stimulated while dSLIM® does not show this off‐target effect (Fig. S7),

The CG‐dependent activation of TLR9 negative cells within PBMC by dSLIM® is probably mediated by IFN‐α. Co‐incubation of dSLIM‐activated PBMC with the vaccinia virus protein B18R, that is known to complex type I interferon 36, abolishes the secretion of IP‐10 as well as the activation of monocytes, NK cells, and NKT cells (Fig. 3). Blocking of type I interferon also impairs the stimulation of PBMC by ODN2216 (see Fig. 3 and others 19), a class A CpG‐ODN with partial PTO‐protection at the termini, known for strong induction of IFN‐α, whereas the ProMune® effects depicted in Figure 3 are hardly affected.

**Figure 4:**
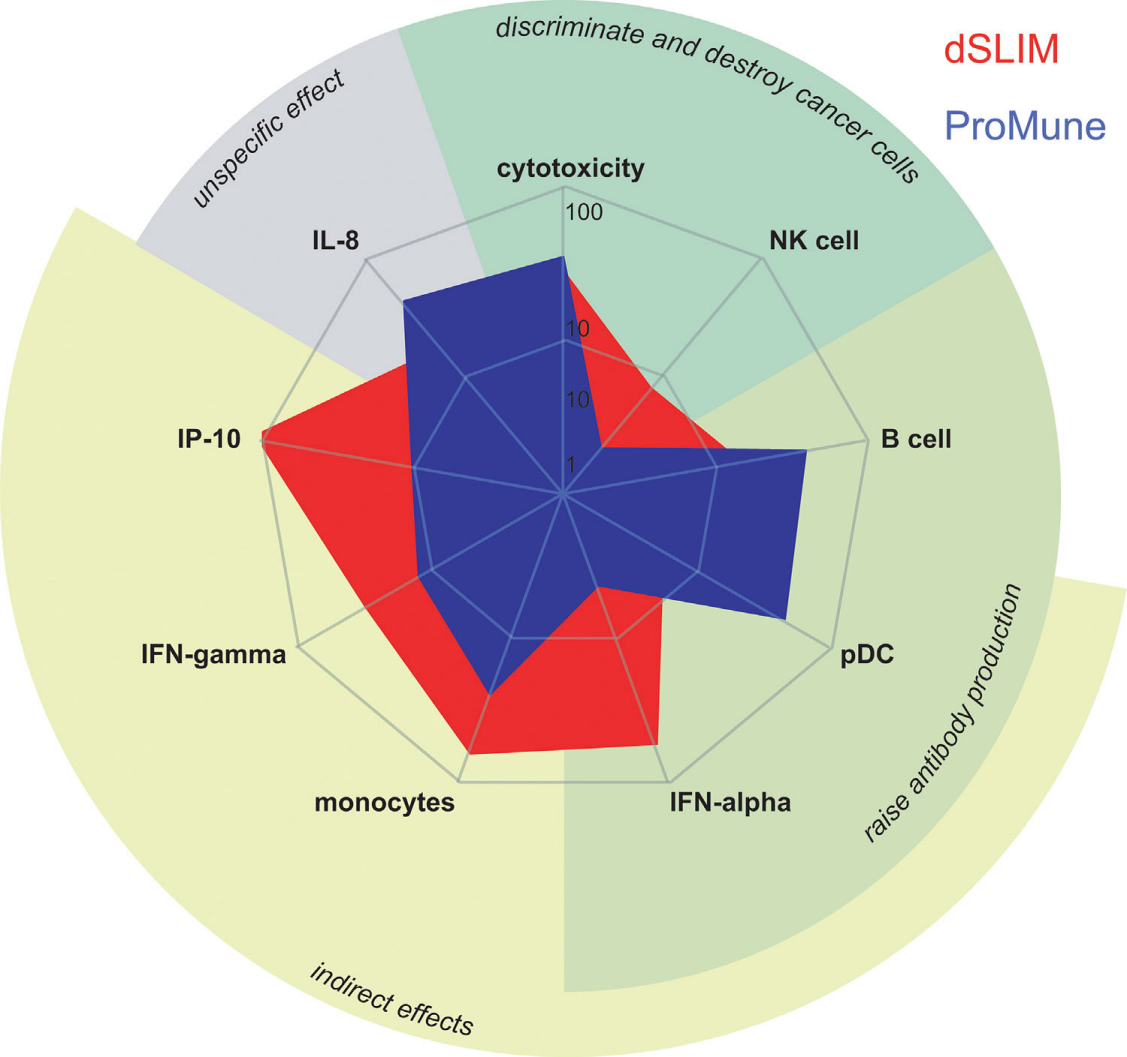
Overview of cancer relevant effects of dSLIM® and ProMune®. Functional parameters of dSLIM® or ProMune® are displayed as geometric means of the best effect each immunomodulator elicited in several independent assays performed with increasing DNA concentrations (partially used for concentration‐effect‐curves shown in Fig. 2) as well as the specific cytotoxicity at effector:target ratio 10:1 listed in Table 1. Due to the different ranges of the nine effects displayed, data were medium‐corrected and scaled as follows: cytotoxicity − 1 × specific cytotoxicity; NK cell – 1 × frequency of CD69 upregulation in NK cell population (*n* = 4); B cell − 1 × upregulation of CD86 on B cells indicated as MFI (*n* = 8); pDC − 0.01 × upregulation of CD86 on pDC indicated as MFI (*n* = 6); IFN‐α − 0.1 × concentration in supernatant from PBMC indicated as pg/mL (*n* = 11); monocyte − 1 × upregulation of CD86 on monocytes indicated as MFI (*n* = 8); IFN‐γ − 0.1 × concentration in supernatant from PBMC indicated as pg/mL (*n* = 12); IP‐10 − 0.001 × concentration in supernatant from PBMC indicated as pg/mL (*n* = 14); IL‐8 − 0.001 × concentration in supernatant from PBMC indicated as pg/mL (*n* = 12).

The relevant immunological effects of dSLIM® and ProMune®, represented by plasma membrane expression in immune cells as well as the cytokine release induced, are summarized in Figure 4. For each TLR9 agonist the maximal promotable effect according to Figure 2 is depicted meaning that both agonists are compared at different concentrations (for details see legend to Fig. 4). In this best‐of‐scenario, dSLIM® more uniformly and often more pronounced induces tumor fighting parameters than does ProMune® what may have caused the different outcomes of their clinical studies 26, 28.

### Linearization and PTO‐modification of dSLIM® loop segment

While dSLIM® and ProMune® both are described as TLR9 agonists and comprise CG‐motifs, they clearly differ in molecule parameters (see Fig. 1a). Pronounced differences are in size (116 nucleotides in dSLIM® vs. 24 nucleotides in ProMune®), conformation (covalently closed, dumbbell‐shaped dSLIM® vs. linear ProMune®), nucleotide sequence, chemical modification of DNA backbone (all genuine phosphorodiester bonds in dSLIM® vs. 23 PTO bonds in ProMune®), number of CG‐motifs (a dimer of three in dSLIM® vs. a monomer of four in ProMune®), as well as relative positioning and sequence environment of CG‐motifs. In order to analyze the structure‐function relationship of dSLIM® that may be responsible for its non‐toxic and improved immunoactivation, we transformed the single‐stranded loop segment with CG‐motifs of dSLIM® into a ProMune®‐like structure. The 30 nucleotides of the loop sequence were synthesized as linear ODN with PTO linkages, to result in a molecule with **l**inearized and fully PTO‐**m**odified **l**oop **s**equence (LMLS‐PTO).

**Figure 5:**
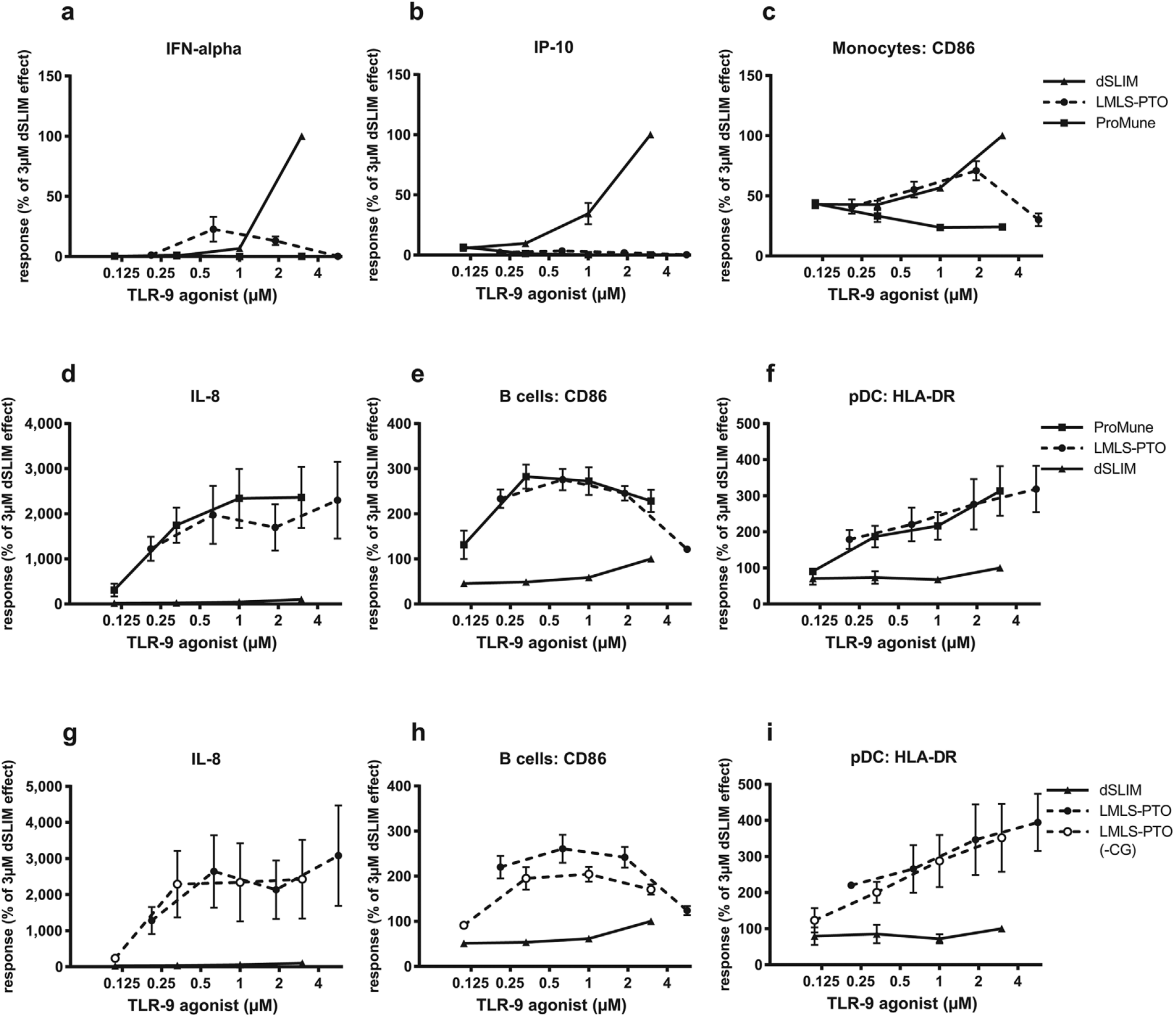
PTO‐modified CpG‐ODN and their CG‐free variants. PBMC were treated with the TLR9 agonists dSLIM®, ProMune®, the linearized, fully PTO‐modified dSLIM® loop (LMLS‐PTO), or the appropriate non‐CG control ((LMLS‐PTO(‐CG)) for 48 h at the indicated concentrations. Cytokine levels in the supernatants were determined by ELISA. Cells were stained with antibodies against lineage‐ and activation markers and analyzed by flow cytometry as described in Materials and Methods section. MFI of activation markers within the cell population are shown. For each parameter, the analyzed effect at the investigated concentrations was normalized to the effect observed at a dSLIM® concentration of 3 μM within the test series. Statistical evaluation for the differences between LMLS‐PTO and the other molecule was calculated for stimulator concentrations with the most prominent effect in each series using the repeated measures ANOVA with the Fisher's LSD post‐test. (a–f): IFN‐α *n* = 6, LMLS‐PTO (0.6 μM) versus dSLIM® (3 μM) ***, LMLS‐PTO (0.6 μM) versus ProMune® (0.3 μM) *; IP‐10 *n* = 8, LMLS‐PTO (0.6 μM) versus dSLIM® (3 μM) ***, LMLS‐PTO (0.6 μM) versus ProMune® (0.1 μM) NS; monocytes CD86 *n* = 7, LMLS‐PTO (1.9 μM) versus dSLIM® (3 μM) ***, LMLS‐PTO (1.9 μM) versus ProMune® (0.1 μM) **; IL‐8 *n* = 7, LMLS‐PTO (5.7 μM) versus dSLIM® (3 μM) ***, LMLS‐PTO (5.7 μM) versus ProMune® (3 μM) NS; B cells CD86 *n* = 7, LMLS‐PTO (0.6 μM) versus dSLIM® (3 μM) ***, LMLS‐PTO (0.6 μM) versus ProMune® (0.3 μM) NS; pDC HLA‐DR *n* = 5, LMLS‐PTO (5.7 μM) versus dSLIM® (3 μM) ***, LMLS‐PTO (5.7 μM) versus ProMune® (3 μM) NS. (g–i): IL‐8 *n* = 5, B cells CD86 and pDC HLA‐DR *n* = 4; means and SEM are shown.

If separated from its dumbbell‐shaped molecule environment in dSLIM® and stabilized by PTO, the dissolved loop does no longer generate its genuine activation pattern on immune cells. Over a concentration range from 0.1 to 3 μM DNA, secretion of IP‐10 (Fig. 5b) is abolished. Only a marginal induction of IFN‐α secretion is observed at low concentrations of LMLS‐PTO (Fig. 5a). Regarding the efficiency to up‐regulate CD86 on monocytes LMLS‐PTO ranges between dSLIM® and ProMune®. Thereby, LMLS‐PTO shows the functional footprint of ProMune® with a strong secretion of IL‐8 (Fig. 5d) and heavy activation of B cells (Fig. 5e) and pDC in terms of HLA‐DR up‐regulation (Fig. 5f). This indicates that activation features of ProMune® on immune cells can be mimicked by a CpG‐ODN with unrelated base sequence and reduced number and different spacing of CG‐motifs, if the DNA backbone is completely protected by PTO modifications.

With a new set of molecules these new functions of dSLIM‐loops in ProMune® form, LMLS‐PTO, were analyzed for TLR9 specificity. If CG‐motifs in LMLS‐PTO are mutated into a TLR9 irrelevant GC base sequence, IL‐8 secretion (Fig. 5g) and HLA‐DR up‐regulation on TLR9 positive pDC in PBMC (Fig. 5i) are unaffected and remain at the same level. Only a partial CG‐dependency was detected for the up‐regulation of CD86 on B cells (Fig. 5h). This stimulation of immune cells by the CG‐free variant of LMLS‐PTO resembles the off‐target effects of the PTO‐modified CpG‐ODN ProMune® (see Fig. S1).

**Figure 6:**
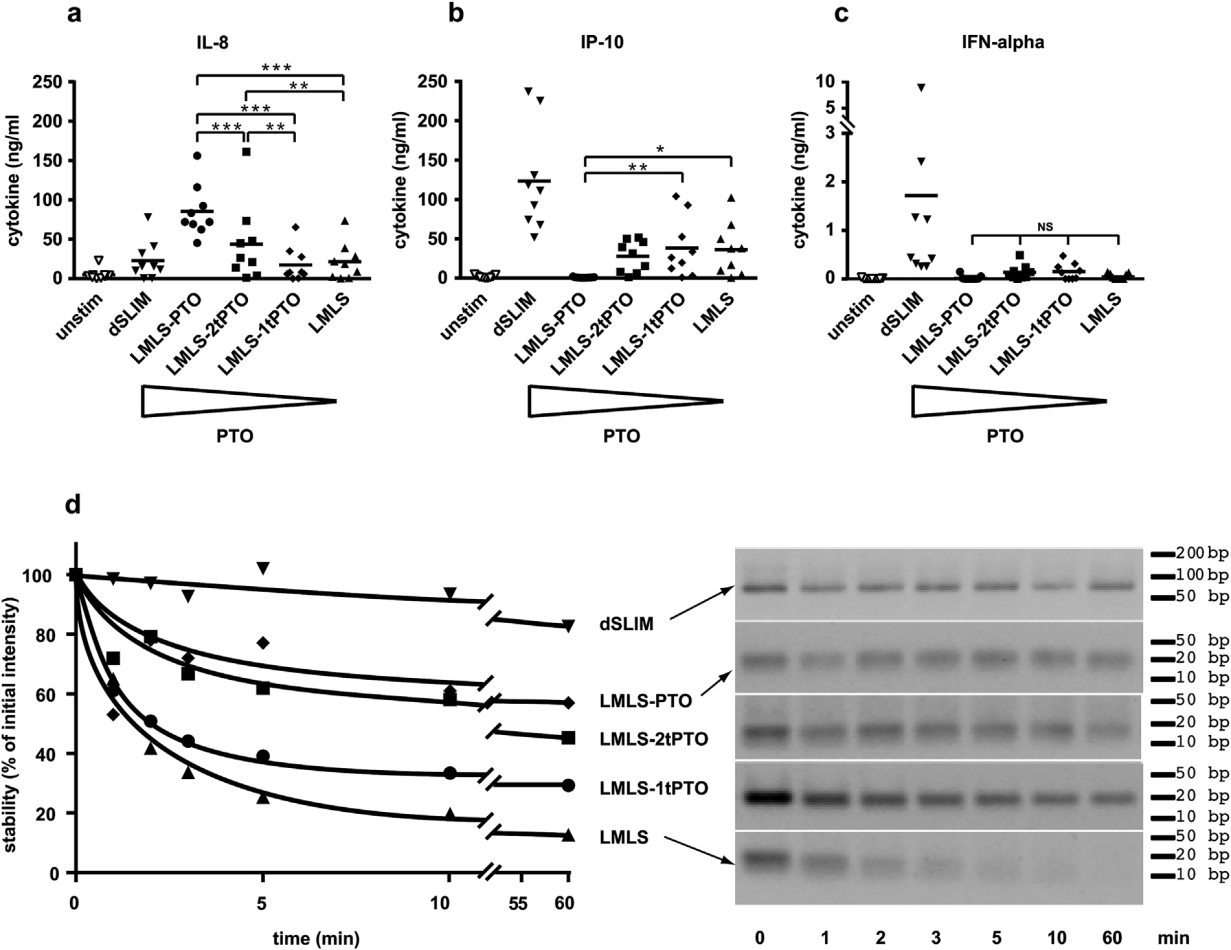
PTO‐dependent features of LMLS. Four molecules comprising the linearized dSLIM loop sequences (LMLS) with different fractions of PTO‐modified nucleotides were generated: LMLS‐PTO:fully PTO‐modified; LMLS‐2tPTO: two PTO‐modified nucleotides at both the 5′‐ and 3′‐end, each; LMLS‐1tPTO: one PTO‐modified nucleotide at both the 5′‐ and 3′‐end, each; LMLS: without any PTO modification. (a–c) PBMC were treated with the indicated activators at final concentrations of 3 μM or medium alone for 48 h. Statistical evaluations are shown for the differences between the LMLS‐molecules. Levels for IL‐8 (a), IP‐10 (b), and IFN‐α (c) in the supernatant were determined by a bead‐based multiplex immunoassay or ELISA (*n* = 9, means are shown, *P < 0.05, **P < 0.01, ***P < 0.001, NS, not significant; repeated measures ANOVA, Fisher's LSD test). (d) LMLS molecules and dSLIM® were subjected to exonuclease treatment (0.22 U of T7 DNA‐polymerase per microgram DNA; 71 ng/μL DNA) for the indicated times and separated on a 3% agarose gel; after staining with ethidiumbromide fluorescence images were taken (right panel) and quantified using the software Quantity One Basic (Bio‐Rad Laboratories, München, Germany). Fluorescence intensities were normalized to the intensity of the corresponding sample at time = 0 min (left panel). One representative out of five similar experiments is shown.

This dominant CG‐independent immunoactivation by PTO‐ODN increases with the fraction of PTO bonds building the linear ODN as shown for the proportional increase of IL‐8 secretion (Fig. 6a). The different contributions of structure and chemistry in the immunomodulator become evident in IP‐10 secretion: the dSLIM® dependent IP‐10 secretion (Fig. 6b) is greatly reduced if the loop nucleotides are no longer in their dumbbell‐shaped structural environment (LMLS). It further diminishes the more genuine phosphodiester‐linked nucleotides are replaced by PTO‐modified nucleotides in the linear ODN molecule. If all loop nucleotides are PTO‐modified (LMLS‐PTO), ODN does no longer induce any IP‐10 secretion. The switch from dSLIM® to linear constructs is most striking for the triggered IFN‐α (Fig. 6c) release: LMLS incubation does no longer induce any detectable IFN‐α release from PBMC; partial PTO‐modification of the molecule has no further measurable effect. It might be conceivable that the observed inefficacy of LMLS with respect to IFN‐α release is the result of a rapid degradation of linear ODN, due to its exonuclease sensitivity. However, the terminal PTO stabilized LMLS molecules do not induce any detectable IFN‐α secretion, either. Alternatively, the unprotected LMLS molecule may induce some low‐level IFN‐α secretion with all molecules already consumed at the time of sample analysis. The latter interpretation is supported by the observed LMLS‐dependent IP‐10 release that may be induced from monocytes by the synergistic action of IFN‐α and IFN‐γ at low concentration 37.

The stabilizing effects of the covalently closed DNA structure in dSLIM® and of PTO in linear ODN are demonstrated in Figure 6d during a 1 h‐exposure of molecules to the 3′‐exonuclease activity of T7 DNA‐polymerase. Under in vitro conditions, optimized for exonuclease activity, LMLS is almost completely degraded within 5 min, while terminal PTO bonds protect the molecule to variable extend: stability of molecules increases with the number of phosphodiester bonds protected by PTO‐modifications at the 3′ end of the ODN, with fully PTO‐protected ODN (LMLS‐PTO) being the most stable among linear ODN. Conformation‐stabilized dSLIM® remained intact over the complete time frame recorded. Actually, in the absence of any open DNA termini, dSLIM® molecules resisted T7 DNA‐polymerase attack for more than 23 h (not shown). Pharmacokinetic parameters (T_1/2_) obtained from clinical trials with cancer patients 38 and healthy volunteers (Table S1) suggest a reasonable in vivo stability of dSLIM against serum nucleases although these data were not corrected for renal clearance. Corresponding half‐times of ProMune® are lower 39.

### Transfer of ProMune® into a dSLIM‐defined dumbbell structure

**Figure 7:**
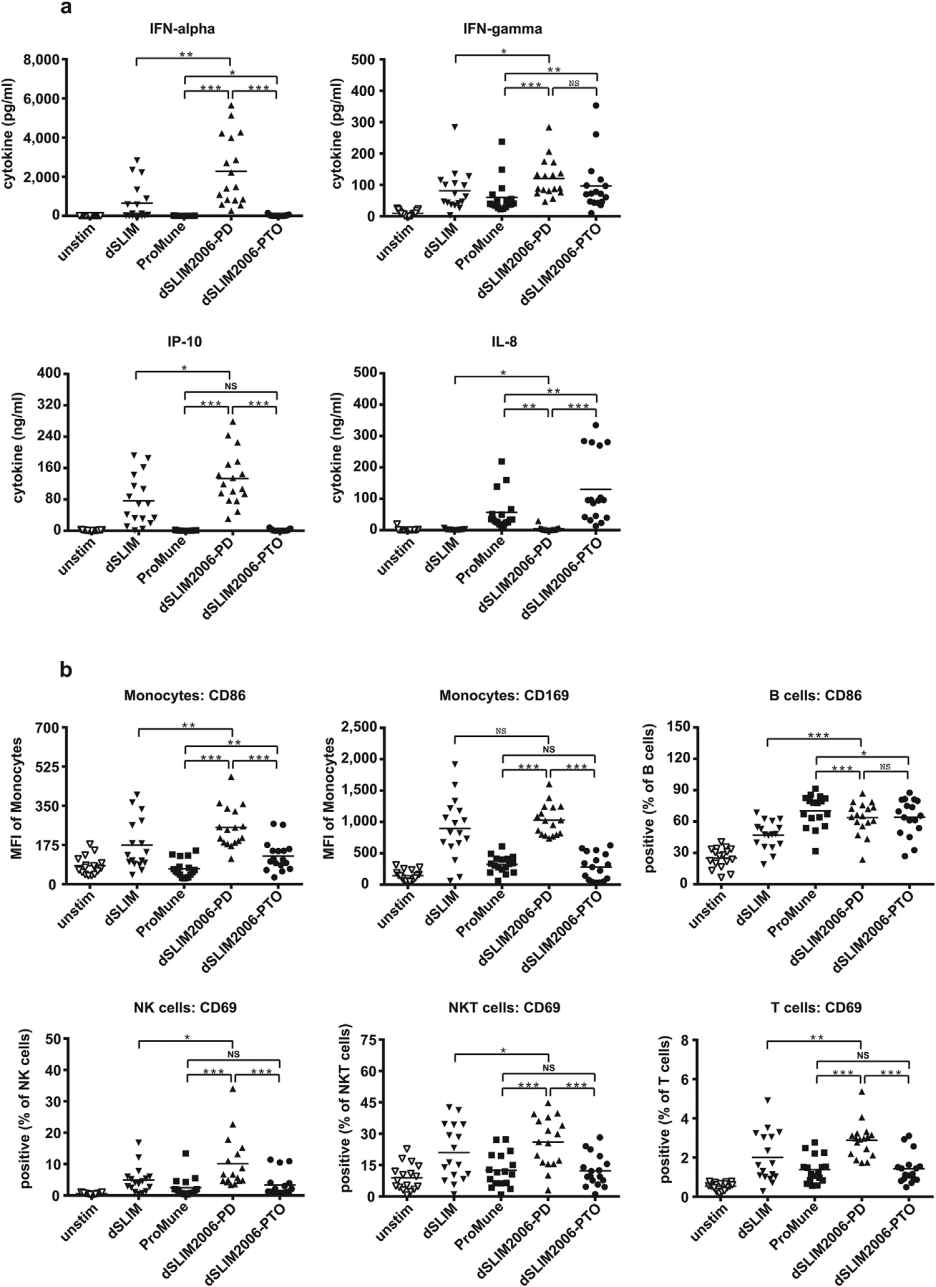
Activation profiles of dSLIM:ProMune chimeras. PBMC were treated with the indicated molecules at final concentrations of 3 μM or with medium alone for 48 h. dSLIM2006‐PD comprises the base sequence of ProMune® in both loop structures of dSLIM® linked by phosphodiester bonds; dSLIM2006‐PTO comprises the base sequence of ProMune® in both loop structures of dSLIM® linked by PTO bonds. Statistical evaluations for the comparisons between both dSLIM2006 molecules and ProMune® as well as between dSLIM2006‐PD and dSLIM® are shown. (a) Cytokine secretion: cytokine levels in the supernatants were determined by a bead‐based multiplex immunoassay or ELISA (IFN‐α, IFN‐γ, IP‐10, IL‐8 *n* = 17, means are shown, *P < 0.05, **P < 0.01, *** p < 0.001, NS, not significant; repeated measures ANOVA, Fisher's LSD test). (b) Cellular activation pattern: cells were stained with antibodies against lineage‐ and activation markers, and analyzed by flow cytometry as described in Materials and Methods section. Frequencies or MFI of activation markers within the cell populations are shown (*n* = 17, means are shown, *P < 0.05, **P < 0.01, ***P < 0.001, NS, not significant; repeated measures ANOVA, Fisher's LSD test).

While dSLIM® loses relevant cancer fighting immunological activities and gains CG‐independent effects when it's active loop sequence was converted into a ProMune‐like conformation (linearization) and chemistry (PTO modified backbone), we also investigated functional changes of ProMune® if adapted to a dSLIM‐like conformation (dumbbell) and chemistry (natural backbone). Therefore, we changed part of the dSLIM® loop sequences to match the base sequence of ProMune® while conserving the dSLIM® stem and overall dumbbell conformation. The resulting molecule dSLIM2006‐PD (see Fig. S5) displays the prevalent dSLIM® profile, like high IFN‐α, IFN‐γ, and IP‐10 secretion (Fig. 7a), and efficacious monocyte, NK cell, NKT cell, and T cell activation (Fig. 7b). In addition, dSLIM2006‐PD shows good CD86 induction in B cells. Interestingly, if the loop nucleotides were PTO‐modified (dSLIM2006‐PTO), relevant dSLIM‐specific functions in dSLIM2006 are blunted, while an undesirable trigger to release IL‐8 (see Fig. 7a) shows up. All newly gained effects of dSLIM2006‐PD that are missing in dSLIM2006‐PTO (e.g., secretion of IFN‐α and IP‐10 as well as activation of monocytes and NK cells) are strictly dependent on CG‐motifs (Fig. S2 for cytokine secretion, Fig. S3 for cell surface markers). In contrast, the residual immunoactivation profile of dSLIM2006‐PTO, in Figures S2 and S3 shown for IL‐8 secretion and the activation of B cells, respectively, is mainly unaffected by the presence of CG‐motifs in the loops.

## Discussion

Using several different in vitro readout systems, we compare the functions of two TLR9 agonists in clinical development for cancer therapy, dSLIM® and ProMune®, differing in sequence, chemical composition, and structure (Figs. 1 and 2). In human PBMC, dSLIM® shows a superior cytokine secretion and cellular activation pattern and addresses all relevant regulators and effectors of an anti‐tumor response: compared to ProMune® 3 μM dSLIM® evoked more than 40‐fold higher secretion of IFN‐α, a central anti‐cancer cytokine regulating innate and adaptive immune responses 40; dSLIM® evoked almost 100‐fold better secretion of IP‐10, a potent angiostatic factor 41; and dSLIM® evoked a 100% better stimulation of IFN**‐**γ‐secretion than ProMune®, the key activator of NK cells, NKT cells, and cytotoxic T cell responses 42. Furthermore, regarding up‐regulation of CD86, dSLIM® leads to a fourfold better activation of monocytes including secretion of immunomodulatory cytokines (IP‐10) and processing of tumor‐associated antigens 43, 44; compared to ProMune®, dSLIM® leads to almost fourfold better activation of NK cells as well as a twofold better activation of NKT cells, both representing effector cells directly attacking tumor cells 45. The different extent dSLIM® and ProMune® express these qualities are characteristic for each of the two TLR9 agonists and are appellative even if dSLIM® and ProMune® are compared at their individual most effective concentrations (see Fig. 4): dSLIM® displays a multifaceted profile for the relevant anti‐cancer features while ProMune® performs well only for some activities with the most pronounced (induction of IL‐8 secretion) being essentially cancer promoting.

The stronger and broader activation of innate immune cells by dSLIM® is most likely due to the better activation of the IFN‐α pathway 17. This is corroborated by the impaired activation of PBMC to secrete cytokines after blocking of type I interferon (see Fig. 3) and the impressive up‐regulation of CD169 on monocytes within PBMC by dSLIM® 25. The expression of CD169 on monocytes has recently been shown to be part of a type I IFN response 34, 35. The mode of action of dSLIM® via the induction of the IFN‐α‐pathway was also strengthened by the in vivo observation of up‐regulation of CD169 in peripheral blood monocytes of patients from the IMPACT trial after s.c. application of dSLIM®, the active ingredient of Lefitolimod (MGN1703) 26. Beside the innate immune response, IFN‐α also regulates the adaptive arm of anti‐tumor response as was recently shown for the activation of CD8‐α^+^ dendritic cells cross‐presenting antigens to cytotoxic T cells 46, 47.

On the other side, ProMune® triggers a fivefold higher secretion of IL‐8, which rather promotes tumor growth through angiogenesis, and a threefold better maturation of pDC and stronger activation of B cells (indicated by CD86 up‐regulation, see Fig. 1d) while dSLIM® directly activates this cell population only to a minor extent. Interestingly, this low performance is partially compensated in the context of other peripheral blood mononuclear cells where dSLIM® at 20 μM induced CD86 expression up to 2/3 of the level that ProMune® induced on B cells at 0.2 μM (see Figs. 2i and 4). It is well conceivable that B cells are synergistically activated by pDC‐released IFN‐α and cell‐cell‐contact 48 in PBMC representing an extension of dSLIM® functional profile beyond the direct cellular effects; the isolated IFN‐α effect seems not to be sufficient to activate B cells beyond the direct, TLR9‐mediated dSLIM® effect on B cells (see Fig. 3h). ProMune® also better maturates pDC albeit—in contrast to B cell stimulation—dSLIM®'s direct effect on CD80‐upregulation in pDC is already remarkable and significant. Our results obtained with ProMune® regarding the strong maturation of and weak IFN‐α secretion from pDC as well as the heavy activation of B cells are in line with findings of other authors 19, 20, 21, 49.

Beside the induction of surface markers on immunorelevant cells, dSLIM® activation leads to the secretion of IP‐10 that is known for its positive impact on anti‐tumor response by its angiostatic properties 41. In contrast, ProMune® does only marginally stimulate IP‐10 release, but strongly the secretion of IL‐8, another important mediator of the innate immune response. However, in cancer treatment, IL‐8 is less appreciated owed to its pro‐angiogenetic effects 50.

While ProMune® activates most immune cells under investigation at concentrations significantly lower than dSLIM®, it requires variable concentrations for optimal activation of different immune cells. In the majority of tested features, ProMune® does not match effect intensities of dSLIM®. The exception is NK cell‐mediated cytotoxicity that at low concentrations of ProMune® somewhat overcomes that evoked by dSLIM. This response was unexpected as the up‐regulation of CD69 is considerably stronger by dSLIM® than by ProMune®. However, the induction of NK cell cytotoxicity can also be triggered by activated pDC and thereby only partially depends on IFN‐α 51, 52 while CD69 up‐regulation on NK cells strictly depends on IFN‐α (see Fig. 3 and 51). Therefore, by the stronger maturation of pDC ProMune® may induce cytolytic NK cell activity at low concentration of IFN‐α. However, at higher concentrations ProMune® NK cell‐mediated cytotoxicity declined as it was observed for most of the other immunological markers what argues for a narrow effective dose range of ProMune®. With the exception of IP‐10 release, the effects exerted by dSLIM® are constantly rising or plateau with increasing agonist concentrations. Throughout the cancer relevant cytokines depicted in Figure 2a–c, the maximal amounts released by optimal concentrations of ProMune® are significantly lower than responses evoked by highest concentrations of dSLIM® applied. In summary, the onset of action and the recorded highest response for every readout parameter in our set‐up was caused by dSLIM® at equal or higher concentrations compared to ProMune®, but steadily built up over a concentration range of at least two orders of magnitude. In contrast, ProMune® induced release of relevant cytokines over a concentration range of only one order of magnitude and declined thereafter indicating some general/broad cytotoxic effect of ProMune® toward immunomodulatory cells. Nevertheless, a direct toxic effect of ProMune® could not be demonstrated by the induction of apoptosis or necrosis in an immortalized mouse macrophage cell line (not shown). Together with activation of pDC and B cells by ProMune® and the decline at higher agonist concentrations, these data point to a small protective index for ProMune® that obviously could not be controlled in vivo in the aborted clinical study 28.

Besides other stimuli, IL‐8 secretion seems to be triggered by PTO modifications in the commonly used TLR9 agonists. All molecules with substantial portion of PTO nucleotides investigated here did stimulate IL‐8 release: ProMune® and its corresponding non‐CG control (see Fig. S1), the single stranded dSLIM‐derived loops after conversion into a PTO‐protected linear CpG‐ODN (LMLS‐PTO) (see Fig. 5d), as well as the CG‐motif free variant of LMLS‐PTO (see Fig. 5g). Notably, the IL‐8 secretion induced by ProMune® as well as by the LMLS‐PTO variants is independent from the proposed stimulatory PyNTTTTGT motif 11, 53. The requirement of PTO modifications for non‐CpG ODN to exhibit some stimulating properties has already been observed in different experimental setups 53, 54 with a strong activation of B cells, but a lack to release Th1‐relevant cytokines like IFN‐α, IFN‐γ, and IP‐10 or to induce a Th2‐like immune response in vivo 54. The PTO effect is corroborated by the positive correlation between evoked IL‐8 secretion and the amount of PTO bonds per molecule (see Fig. 6). While IL‐8 secretion increases with PTO content of stimulator, inverse correlation is observed for IP‐10 secretion. Due to their CG‐independency, we assume that these off‐target effects of PTO‐modified ODN are induced at sites different from TLR9, either on TLR9‐positive or TLR9‐non expressing cells (shown for isolated monocytes in Fig. S7).

Despite the known limitations in transferring in vitro results onto living systems and even into patients clinical response, the presented profiles of dSLIM® and ProMune® correlate surprisingly well with clinical data. Together with the manifest in vivo efficacy of dSLIM®, our studies show that the used setups to profile dSLIM® in vitro functions yield results that directly translate into clinical outcome. The repeated subcutaneous applications of dSLIM® in the IMPACT study apparently differ from the single addition of dSLIM® to freshly isolated PBMC in cell culture medium. Nonetheless, activation of immune cells (especially CD169 on monocytes, but also activation of pDC and NK cells 26) and increase in secreted IP‐10 (M. Schroff, personal communication, 2015) were detectable in both systems. Other readouts from PBMC could not be applied in the clinical study due to rapid consumption of secreted cytokines (especially IFN‐α) and limitations in tissue sampling due to ethical concerns.

Further systematic comparison of dSLIM® with the single‐stranded, PTO‐modified ProMune® in human PBMC cultures shows that—besides chemistry (PTO modification) and CG‐motifs—also the structure of the agonist determines its function in immune cell activation: the CG‐dependent part of the prominent B cell‐stimulating feature of ProMune® can be added to the favorable anti‐cancer profile of dSLIM® by introducing the base sequence of ProMune® into the phosphodiester‐bonded loops of dSLIM®. Thereby, in dSLIM2006‐PD the IFN‐α secretion and the subsequent broad activation of the innate immune system by dSLIM® are preserved, and good activation of B cells is added (see Fig. 7). Apparently, the unique dumbbell‐shaped conformation of dSLIM® is a prerequisite for a stable TLR9‐triggered IFN‐α release and may be—together with the CG‐motifs of the agonist—an additional feature to effectively stimulate the IRF7‐signaling branch of TLR9 triggered pathways.

In interesting approaches, IFN‐α releasing activity was conferred to class B (or K‐type) ODN by multimerization into nanoparticles 55 or more recently by mixing with the HIV‐derived peptide Tat_(47–57)_ to form stable nanorings 56. Features common to these multimeric structures and dSLIM® are several CG‐motifs multimerized in a flexible spacing. In contrast to the nanoparticles or nanorings with unknown final composition of the components, the monomeric dSLIM® is well defined by three CG‐motifs in dimer constellation and devoid of further additives. Additives like nanoparticles or peptides may induce toxicities or unwanted immune responses, and therefore can hamper their clinical application. In contrast, dSLIM® molecules (Lefitolimod, MGN1703), exclusively consisting of natural DNA and applied without any additive, showed an excellent safety profile in a recent phase II clinical trial for metastatic colorectal carcinoma 26. While dimerization may qualify a TLR9 agonist to strongly induce IFN‐α secretion in pDC (and subsequently IP‐10 release from monocytes), individual base composition, sequence, and/or number of CG‐motifs appear to be relevant to activate B cells. The dSLIM® and particularly dSLIM2006‐PD efficiently combine sequence parameters with structural parameters to form efficient TLR9 agonists with highly efficient anti‐tumor activity. Certainly, dSLIM® structure does not render every poor immunomodulatory CpG‐ODN into a potent anti‐cancer vaccine. For instance, the favorable anti‐cancer footprint of dSLIM® can be masked or even reverted if a non‐natural PTO backbone is used to build up this molecule (dSLIM2006‐PTO). We cannot distinguish if PTO‐modified nucleotides impact the overall dSLIM® structure or just change interaction with proteins, including TLR9. However, the pronounced increase of IFN‐α induction by PTO‐free ODN2216 (see Fig. S4) and the complete deficit of fully PTO‐modified ODN2216 (ODN2216‐PTO) to release any detectable IFN‐α argue for the latter scenario. Furthermore, activation of NK cells 57 and secretion of IFN‐γ 20 in the context of human PBMC require a central phosphorodiester‐backbone in the stimulation by TLR9 agonist. Irrespective of the nature of molecular interaction PTO modifications in ProMune® mediate considerable off‐target‐effects that could be avoided if the dumbbell structure of dSLIM® is used to protect the ODN from nucleolytic degradation. Even other immunological active sequences than that from ProMune® may profit from a dSLIM‐like conformation to enhance their efficiency and broaden their functional profile.

## Authors’ Contributions

CK and KK designed the experiments, interpreted, and plotted the results and wrote the paper. NG, SJ, JS, and LS performed experiments. BW and MS conducted the conceptual design of the studies and reviewed the manuscript. BW invented dSLIM.

### Conflict of interest

KK, JS, LS, NG, SJ, CK, and MS are employees of Mologen AG. BW consults Mologen AG and receives funding from Mologen AG. All authors have commercial interest in the therapeutic development of dSLIM®, partially hold shares from Mologen AG but have no additional financial interests.

## Supporting information

Additional supporting information may be found in the online version of this article at the publisher's web‐site.

**Figure S1**. CG‐dependency of ProMune® effects.

**Figure S2**. CG‐dependency of dSLIM2006 effects on immune cells: cytokine secretion.

**Figure S3**. CG‐dependency of dSLIM2006 effects on immune cells: activation markers on cells.

**Figure S4**. Influence of the PTO‐content of ODN2216 on IFN‐α secretion.

**Figure S5**. Sequence and proposed structure of dSLIM2006.

**Figure S6**. Time course of PBMC activation by dSLIM® and ProMune®.

**Figure S7**. Activation of TLR9 negative monocytes and CG dependency.

**Table S1**. Pharmacokinetic parameters of dSLIM®, the active ingredient of Lefitolimod (MGN1703).
