# Supplementary material for: Distinct immunological activation profiles of dSLIM® and ProMune® depend on their different structural context
Source: Immun Inflamm Dis. 2016 Oct 18;4(4):446–62. doi: 10.1002/iid3.126 (PMC5134728; doi:10.1002/iid3.126)
Supplement: Supplementary file 8 — Table S1. Pharmacokinetic parameters of dSLIM®, the active ingredient of Lefitolimod (MGN1703). [file IID3-4-446-s008.docx]

**SUPPLEMENT FIGURE LEGENDS**

**Fig. S1**: **CG-dependency of ProMune® effects.**

PBMC were treated with ProMune**®** or ProMune(-CG) at the indicated final concentrations for 48 h.

Cells were stained with antibodies against lineage- and activations markers and analyzed by flow cytometry as described in Materials and Methods. For each analyte the ratio between the MFI of stimulated cells and the MFI of cells incubated in medium alone was calculated (B cells CD86: 9.05-15.4; monocytes CD86: 83.8-91.4). Means and SEM resulting from 5 individual experiments are shown.

Cytokine levels in the supernatants of cells were determined by ELISA. For each cytokine the ratio of cytokine levels of stimulated cells and cells incubated in medium alone (312-2981 pg/ml) was calculated. Means and SEM resulting from the stated numbers of individual experiments (n=5) are shown. The multiple t-test was used to qualify the differences between ProMune**®** and ProMune(-CG) at equal concentrations applied (* p<0.05, ** p<0.01).

**Fig. S2: CG-dependency of dSLIM2006 effects on immune cells: cytokine secretion.**

PBMC were treated with the indicated molecules at final concentrations of 3 µM or with medium alone for 48 h. The dSLIM2006-PD comprises the base sequence of ProMune® in both loop structures of dSLIM®, linked by phosphodiester bonds; dSLIM2006-PD(-CG) relates to the dSLIM2006-PD with each of the 3 CG-motifs in both loops mutated into sequence GC. dSLIM2006-PTO comprises the base sequence of ProMune® in both loop structures of dSLIM®, linked by PTO bonds; dSLIM2006-PTO(-CG) relates to dSLIM2006-PTO with each of the 3 CG-motifs in both loops mutated into sequence GC. Cytokine levels in the supernatant were determined by a bead based multiplex immunoassay or ELISA (n=4; means are shown).

**Fig. S3: CG-dependency of dSLIM2006 effects on immune cells: activation markers on cells.**

PBMC were treated with the indicated molecules at final concentrations of 3 µM or with medium alone for 48 h. Molecules for stimulation are described in the legend of Fig. S2. Cells were stained with antibodies against lineage- and activation markers, and analyzed by flow cytometry as described in Materials and Methods. Frequencies or MFI of activation markers within the cell populations are shown (n=4; means are shown).

**Fig**. **S4**: **Influence of the PTO-content of ODN2216 on IFN-alpha secretion**

PBMC were treated with the indicated molecules at final concentrations of 3 µM or with medium alone for 48 h. The molecule ODN2216 is protected with 2 PTO-bonds at the 5`and 5 PTO bonds at the 3`end is already described in Materials and Methods. ODN2216-PTO and ODN2216-PD present the same base sequence as ODN2216, but comprise the backbone entirely as phosphothioester and phosphodiester bonds, respectively. IFN-alpha levels in the supernatant were determined by ELISA (n=7; means are shown).

**Fig. S5: Sequence and proposed structure of dSLIM2006.**

CG-motifs are depicted in bold.

**Fig.S6: Time course of PBMC activation by dSLIM® and ProMune®.**

PBMC were treated for the indicated times with medium alone or the optimal concentrations of eachTLR9 agonists: 3 µM dSLIM® or 0.2 µM ProMune®. Cytokine levels in the supernatants were determined by ELISA. Cells were stained with antibodies against lineage- and activation markers, and analyzed by flow cytometry as described in MATERIALS AND METHODS. MFI of activation markers within the cell populations are shown. Values of two blood donors are shown as different shades in stacked columns for the treatment with dSLIM® (in red) or ProMune® (in blue).

**Fig.S7: Activation of TLR9 negative monocytes and CG dependency.**

Monocytes were isolated from PBMCs using the CD14 MicroBead Kit, (Miltenyi Biotec). Monocytes were treated with the indicated molecules at final concentrations of 3 µM or with medium alone for 48 h. IL-8 levels in the supernatants were determined by a beadbased multiplex immunoassay or ELISA. Cells were stained with antibodies against activation markers, and analyzed by flow cytometry as described in MATERIALS AND METHODS. MFI of activation markers within the cell populations are shown.

For part A, amounts of IL-8 and up-regulation of surface markers on monocytes were normalized to the medium control.

|  | C_max_ (ng/ml) | T_max_ (hours) | T_1/2_ (hours) |
| --- | --- | --- | --- |
| Median | 166 | 14 | 12,7 |
| Minimum | 53,4 | 8 | 9,36 |
| Maximum | 449 | 16 | 17,9 |

**Table S1: Pharmacokinetic parameters of dSLIM®, the active ingredient of Lefitolimod (MGN1703)**

# 13 healthy volunteers of the clinical trial NCT01982747 (A Phase I Study to Assess Cardiac and General Safety and Pharmacokinetics of 60 mg Lefitolimod, MGN1703) had been injected subcutaneously with 60 mg of Lefitolimod (MGN1703). Serum concentration of dSLIM® was determined after 1, 3, 6, 8, 10, 12, 14, 16, 24, 48, 72, and 96 hours after dosing as described ^1^ with minor modifications. Pharmacokinetic parameters derived from serum levels over time are listed as median with lowest (minimum) and highest (maximum) values analyzed. C_max_, maximum concentration measured in serum over individual time courses; T_max_, time to maximal serum concentration; T_1/2_, half-life of dSLIM®.

^1^ Weihrauch, MR, Richly, H, von Bergwelt-Baildon, MS, Becker, HJ, Schmidt, M, Hacker, UT*, et al.* (2015). Phase I clinical study of the toll-like receptor 9 agonist MGN1703 in patients with metastatic solid tumours. *European journal of cancer* **51**: 146-156.
